# Preparation of Magnetic Nanoparticles via a Chemically Induced Transition: Role of Treating Solution’s Temperature

**DOI:** 10.3390/nano7080220

**Published:** 2017-08-12

**Authors:** Ting Zhang, Xiangshen Meng, Zhenghong He, Yueqiang Lin, Xiaodong Liu, Decai Li, Jian Li, Xiaoyan Qiu

**Affiliations:** 1 School of Physical Science and Technology, Southwest University, Chongqing 400715, China; zhangting07@email.swu.edu.cn (T.Z.); 15552267851@163.com (X.M.); hezhenho@swu.edu.cn (Z.H.); linyq@swu.edu.cn (Y.L.); liuzm@swu.edu.cn (X.L.); 2 State Key Laboratory of Tribology, Tsinghua University, Beijing 100084, China; lidecai@tsinghua.edu.cn

**Keywords:** *γ*-Fe_2_O_3_ nanoparticle, FeCl_2_ solution, temperature, magnetization

## Abstract

Using FeOOH/Mg(OH)_2_ as precursor and FeCl_2_ as the treating solution, we prepared *γ*-Fe_2_O_3_ based nanoparticles. The FeCl_2_ treating solution catalyzes the chemical reactions, dismutation and oxygenation, leading to the formation of products FeCl_3_ and Fe_2_O_3_, respectively. The treating solution (FeCl_2_) accelerates dehydration of the FeOOH compound in the precursor and transforms it into the initial seed crystallite *γ*-Fe_2_O_3_. Fe_2_O_3_ grows epitaxially on the initial seed crystallite *γ*-Fe_2_O_3_. The epitaxial layer has a magnetically silent surface, which does not have any magnetization contribution toward the breaking of crystal symmetry. FeCl_3_ would be absorbed to form the FeCl_3_·6H_2_O surface layer outside the particles to form *γ*-Fe_2_O_3_/FeCl_3_·6H_2_O nanoparticles. When the treating solution’s temperature is below 70 °C, the dehydration reaction of FeOOH is incomplete and the as-prepared samples are a mixture of both FeOOH and *γ*-Fe_2_O_3_/FeCl_3_·6H_2_O nanoparticles. As the treating solution’s temperature increases from 70 to 90 °C, the contents of both FeCl_3_·6H_2_O and the epitaxial Fe_2_O_3_ increased in totality.

## 1. Introduction

Nanotechnology involves the study of matter whose dimensions approximately range between 1 and 100 nm [1]. Nanoparticles are typically defined as tiny solids, whose dimensions do not exceed 100 nm in all the three directions [2]. Magnetic nanoparticles have attracted a lot of interest in the community of researchers, because these tiny particles are extremely useful models for understanding the fundamental aspects of magnetic ordering phenomena in magnetic materials with small dimensions. The findings of these research studies can be used to develop novel technological applications [3,4,5]. In most studies of magnetic nanoparticles, scientists have tried to develop novel synthesis methods [2]. Liquid phase synthesis is one of the most common methods to produce inorganic nanoparticles. Many oxide nanoparticles, including ferrite particles, can be synthesized by co-precipitation. The chemical reactions involved in the synthesis of oxide nanoparticles can be classified into two categories: (i) oxide nanoparticles produced directly and (ii) production of a precursor that is then subjected to further processing, such as drying, calcination, etc. [6]. During the chemical reaction, a new phase is formed that is later subjected to further processing, such as calcination or annealing.

Presently, *γ*-Fe_2_O_3_(maghemite) particles are one of the commonly used ferric oxide particles for their simple synthesis procedures and chemical stability [7]. Maghemite exhibits ferrimagnetic behavior at temperatures lower than 1000 K. Furthermore, it is found in corrosion products, but also in several useful compounds, including proteins [8]. It has many industrial applications: as a drug delivery agent; in nuclear magnetic resonance imaging; in magnetic data storage applications; etc. [7,8,9]. Previous studies have described many novel methods for the preparation of *γ*-Fe_2_O_3_ magnetic nanoparticles, including co-precipitation, gas-phase reaction, direct thermal decomposition, thermal decomposition/oxidation, sonochemical synthesis, microemulsion reaction, hydrothermal synthesis, vaporization-condensation, and sol-gel approach [10,11,12,13,14,15,16,17,18]. In general, the preparation of *γ*-Fe_2_O_3_ by FeOOH transformation is a complex process [19,20] that can be summarized as follows:

$$\alpha \left(\gamma \right)\mathrm{FeOOH}\overset{\text{ dehydration }}{\to }\alpha \left(\gamma \right)-Fe_{2}O_{3}\overset{\text{ reduction }}{\to }Fe_{3}O_{4}\overset{\text{ oxidation }}{\to }\gamma -Fe_{2}O_{3}$$

We have found a new route to synthesize *γ*-Fe_2_O_3_ magnetic nanoparticles. In this method, we synthesize the precursor FeOOH/Mg(OH)_2_ by conducting a chemical co-precipitation method. The resultant hydroxide precursor FeOOH/Mg(OH)_2_ is subsequently treated in the liquid phase with FeCl_2_ solution [21]. During the treatment, Mg(OH)_2_ compound dissolves and the FeOOH undergoes dehydration and transforms into *γ*-Fe_2_O_3_ nanoparticles:

$$\mathrm{FeOOH}/\mathrm{Mg}{\left(\mathrm{OH}\right)}_{2}\overset{\Delta }{\underset{\;\mathrm{in}\;{\mathrm{Fecl}}_{2}\;\mathrm{solution}}{\to }}\gamma -Fe_{2}O_{3}+H_{2}O+Mg^{2+}+OH^{-}$$

This method is known as chemically induced transition (CIT) [22,23]. Under boiling conditions, we could synthesize *γ*-Fe_2_O_3_ nanoparticles coated by FeCl_3_·6H_2_O by ensuring that the concentration of the FeCl_2_ solution was in the range of 0.06–0.25 M [23]. In this experimental study, we adjust the temperature of the treating solution and investigate whether magnetization is dependent on the temperature, and the relevance between magnetization and components.

## 2. Experimental

### 2.1. Preparation Using Chemicals

From China National Medicines Corporation Ltd. (Shanghai, China), we purchased the following analytical reagent (AR) grade chemicals: FeCl_3_·6H_2_O, Mg(OH)_2_·6H_2_O, NaOH, FeCl_2_·4H_2_O and acetone. Since these reagents were of AR quality, we used them without performing further purification. We used only distilled water for performing the preparations of solutions in the experiment.

While performing this CIT method, we categorically divided the preparation process of nanoparticles into two steps: (i) we carried out the well-known method of co-precipitation to synthesize a precursor based on FeOOH; the precursor was wrapped with Mg(OH)_2_. The synthesis of this precursor has been described in detail elsewhere [21]; (ii) we added 5 g of the dried precursor to 400 mL of 0.25 M FeCl_2_ solution. The pH of resultant solution was about 6. Then, the resultant solution was heated to a certain temperature, and then it was refluxed for 30 min in air. After completing the process of heating, we were able to obtain nanoparticles gradually in the form of a precipitate. Finally, we washed the precipitate with acetone and air-dried it in the laboratory. We obtained the samples (1)–(5) by adjusting the temperature of the treating solution to the following respective values: 40, 60, 70, 80, and 90 °C.

### 2.2. Characterization

For precursor and samples (1)–(5), we measured the curves of specific magnetization (*σ*) against field strength (H) using vibrating sample magnetometer (VSM) (HH-15, Nanjing University Instrument Plant, Nanjing, China). After obtaining the measured results of VSM, we performed transmission electron microscopy (TEM) (TEM-2100F, Tokyo, Japan) on all the samples; however, we record particle morphologies only in the following typical sample (1) (treated solution temperature: 40 °C), sample (3) (treated solution temperature: 70 °C), and sample (5) (treated solution temperature: 90 °C), according to the results measured by VSM. Then, we analyzed the crystal structure of samples by X-ray diffraction (XRD) (D/Max-Rc, Rigaku, Tokyo, Japan). We analyzed the bulk chemical species by performing energy disperse X-ray spectroscopy (EDS) in a scanning electron microscopy (SEM) (Quanta-200, FEI, Hillsboro, OR, USA). Finally, we analyzed surface chemical compositions of samples by performing X-ray photoelectron spectroscopy (XPS) (ESCALAB 250 xi, Thermo Fisher Scientific, Waltham, MA, USA).

## 3. Results

**Figure 1:**
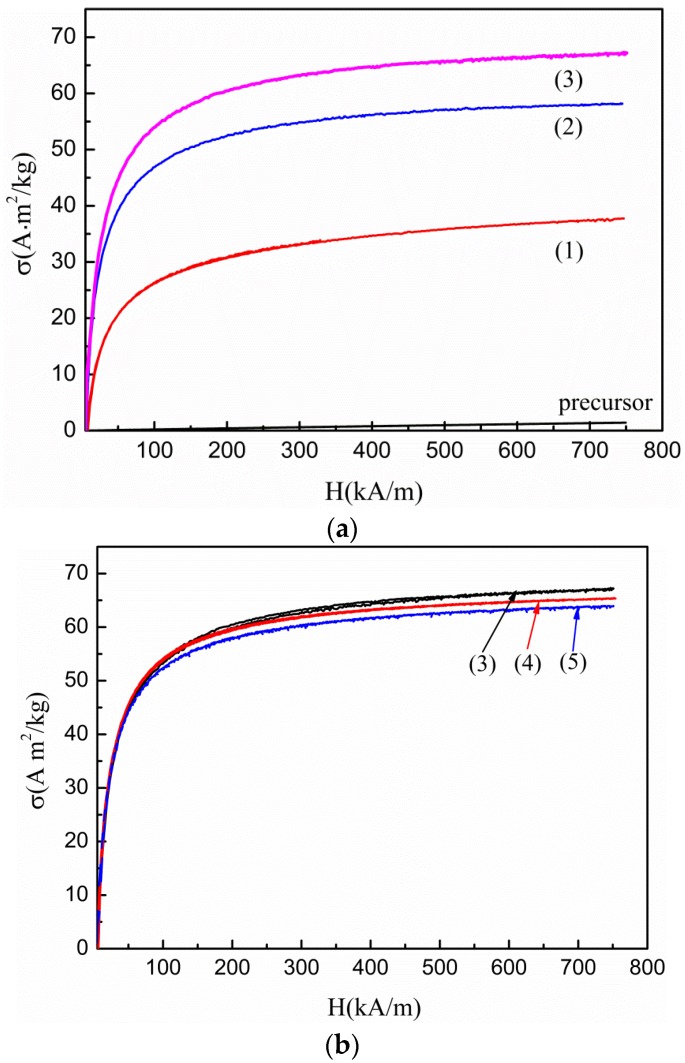
Specific magnetization curves of the precursor and as-prepared samples (1)–(3) (**a**), and as-prepared samples (3)–(5) (**b**).

Figure 1 illustrates the curves representing the plot of *σ* against H for various samples. The precursor was paramagnetic. In contrast, the as-prepared samples exhibited ferromagnetic transition because they were treated with FeCl_2_ solution. Furthermore, the specific magnetization of samples varied non-monotonically with an increase in the temperature of the treating solution: the magnetization (*σ*) values increased drastically as the temperature of treating solution (FeCl_2_) was increased from 40 to 70 °C, but then *σ* values of samples decreased slightly with a further increase in temperature from 70 °C to 90 °C of the treated solution. The specific saturation magnetization (*σ*_s_) of the as-prepared samples was obtained from the plot of *σ* versus 1/H at high field strength [24]. For samples (1)–(5), the *σ*_s_ values are 42.96, 59.33, 70.51, 68.18, and 66.61 A·m^2^/kg, respectively.

**Figure 2:**
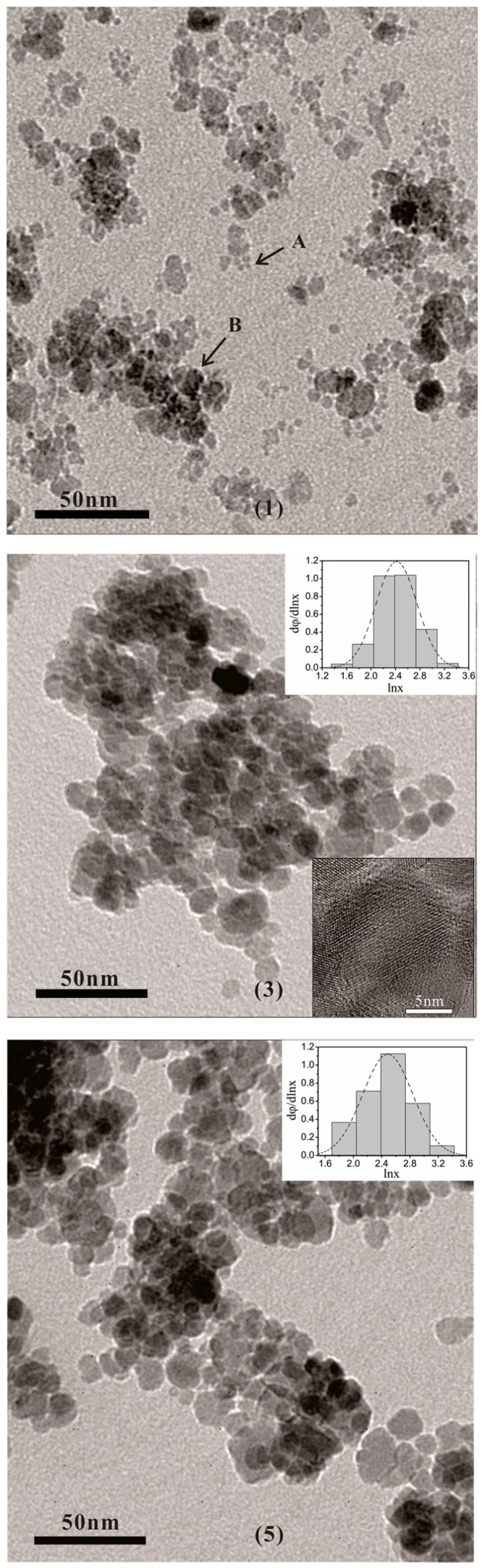
Typical TEM images for samples (1), (3), and (5). The insets are the histograms of the particle sizes for samples (3) and (5), and a High resolution TEM (HRTEM) image for sample (3).

By performing TEM on all the samples, we noted the following observations: the samples (1), (3), and (5) are mostly spherical nanoparticles, with sizes ranging from 2 to 30 nm. Figure 2 illustrates TEM images of the samples. In the case of Sample (1), TEM images clearly depict a small mixture (refer arrow A) and large (refer arrow B) particles. We performed statistical analysis of the results observed for samples (3) and (5) [25]. The histograms of the particle size are illustrated as the insets in Figure 2. Based on the statistical analysis, we inferred the particle size exhibited a log-normal form of distribution. Table 1 presents the median diameter, that is, the most probable value of the particle size d_g_, and the standard deviation ln*σ*_g_. High resolution TEM measurements have been performed in some of the samples (see inset in Figure 2), confirming that the nanoparticles are crystallines.

**Figure 3:**
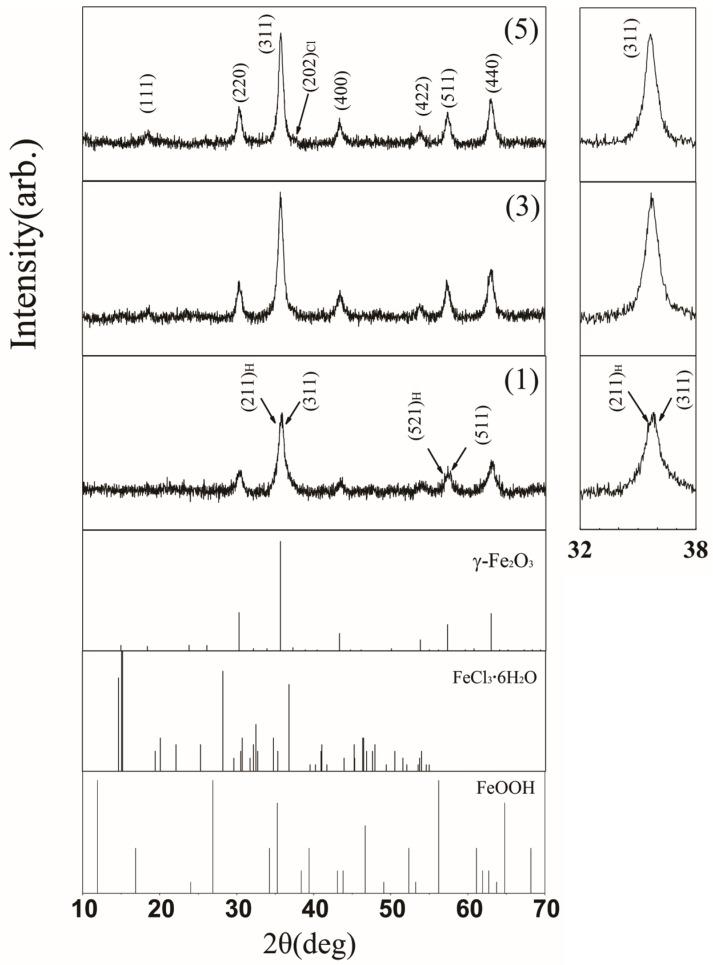
XRD spectra of samples (1), (3), and (5) with (hkl), (hkl)_Cl_ and (hkl)_Mg_ corresponding to *γ*-Fe_2_O_3_, FeCl_3_·6H_2_O and FeOOH phases, respectively.

As shown in Figure 3, XRD patterns reveal that the samples (1), (3), and (5) predominantly contained maghemite (*γ*-Fe_2_O_3_; JCPDS file 39-1346) with traces of hydromolysite (FeCl_3_·6H_2_O; JCPDS file 33-0645). In addition, sample (1) may contain some crystals of iron oxide hydroxide (FeOOH; JCPDS file 13-0157), whose diffraction peak in (211) plane (2*θ* = 35.264) overlapped with the diffraction peak of *γ*-Fe_2_O_3_ in (311) plane (2*θ* = 35.630). This phenomenon is attributed to the broadening of diffraction peaks. For samples (3) and (5), we used Scherrer’s formula to estimate the most probable grain size (d_c_) from the half-maximum width of (311) diffraction peak (*β*) [26,27]. The expression of Scherrer’s formula is as follows: d_c_ = k*λ*/*β*cos*θ*, where k is the coefficient and equals to 0.89 [28], *λ* is the wavelength (Cu K*α* wavelength is 0.1542 nm), and *θ* is the Bragg diffraction angle of (311) plane. Table 1 presents d_c_ values of samples (3) and (5). These values indicate that d_c_ value is almost same for both the samples (3) and (5).

**Figure 4:**
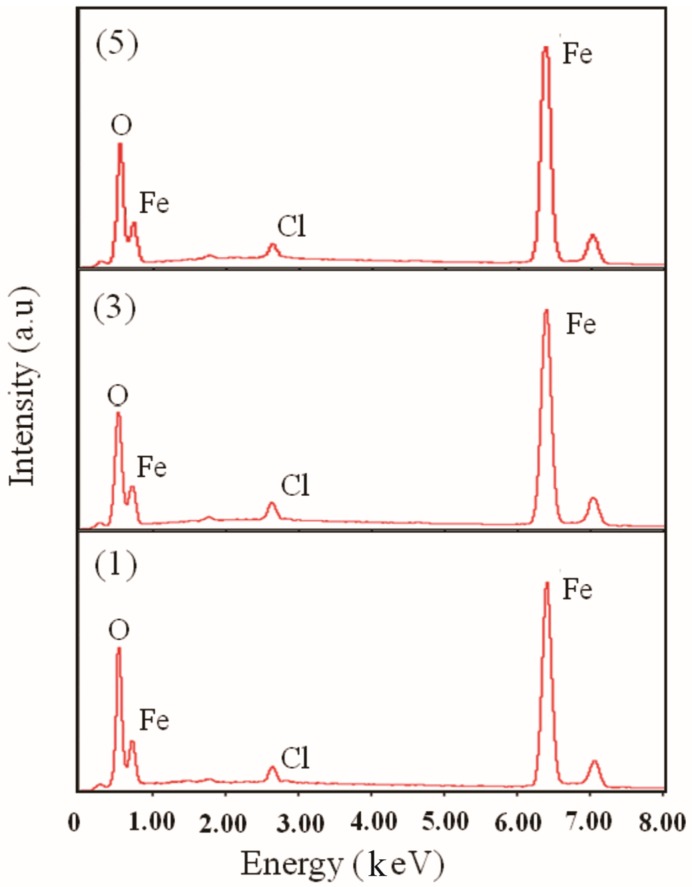
EDS spectra of samples (1), (3), and (5).

By performing energy-dispersive X-ray spectroscopy (EDS), we found that all the three samples contained O, Fe, and Cl, but not Mg and Na. For quantitative analysis, many zones were probed to average the content of each element. Figure 4 illustrates images of typical EDS spectra. Table 2 summarizes the quantitative results of this experiment.

**Figure 5:**
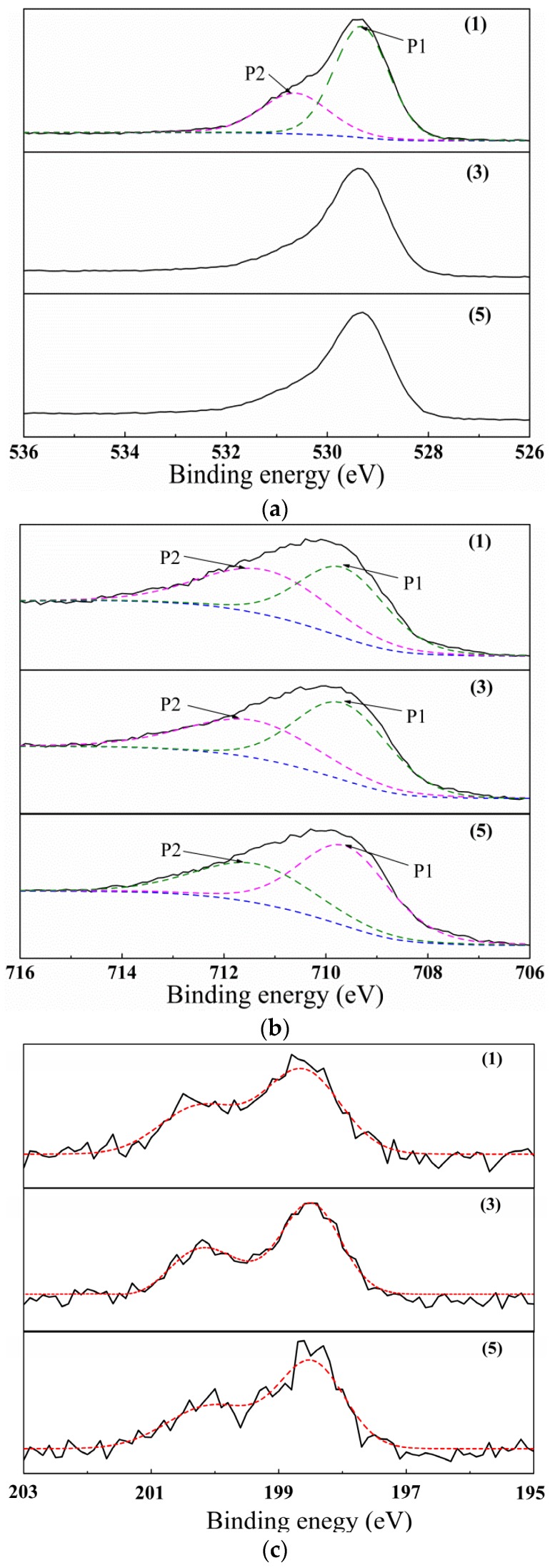
XPS spectra of samples (1), (3), and (5), representing O ls (**a**) Fe 2p_3/2_ (**b**) and Cl 2p (**c**) line regions.

After comparing the results of samples analyzed by XRD and EDS techniques, we conclude that *γ*-Fe_2_O_3_ and FeCl_3_·6H_2_O may be the primary constituents in samples (1), (3), and (5); however, an additional FeOOH compound may be present in sample (1). To examine the surface characteristics of particles, we performed an XPS analysis on the samples. The results of the XPS analysis indicate that the chemical species detected in each sample were the same as those detected by EDS. Table 2 presents a quantitative analysis of results. For sample (1), O1s spectra can be resolved into two peaks: P1 and P2 (See Figure 5a). The P1 peak corresponds to O1s line in samples (3) and (5), which approximately appears at 529.3 eV. Thus, the P1 peak’s energy agreed with the binding energy of O1s in ferric oxide. The P2 peak appears at 530.66 eV, which is same as the binding energy of O1s in FeOOH. Furthermore, the Fe 2p_3/2_ spectra for sample (1), (3), and (5) can be resolved into two peaks: P1 and P2. As shown in Figure 5b, peak P1 corresponds to Fe 2p_3/2_ line of Fe_2_O_3_, while P2 peak corresponds to that of FeOOH and/or FeCl_3_. Table 3 summarizes the results obtained by performing XPS analysis on the samples. Thus, based on the binding energy data, we conclude that *γ*-Fe_2_O_3_, FeCl_3_·6H_2_O and FeOOH were present in sample (1), while *γ*-Fe_2_O_3_ and FeCl_3_·6H_2_O were present in samples (3) and (5).

## 4. Discussion 

Based on the experimental results, it is showed that the magnetization of as-prepared samples varied non-monotonically with an increase in the temperature of treated solution. Combining the results from XRD and XPS, we conclude there could be *γ*-Fe_2_O_3_ and FeCl_3_·6H_2_O phases in all samples and an addition FeOOH phase in sample (1), which is in agreement with our previous work [21]. In addition, it is noticed that the ferrite-like spinel structure, *γ*-Fe_2_O_3_ and Fe_3_O_4_, is difficult to discriminate by XRD due to peak broadening [29] or by XPS because the data are very close (the binding energy of Fe 2p_3/2_ in Fe_3_O_4_ is 710.8 eV). However, Fe_3_O_4_ is not stable and is sensitive to oxidation [9]. It was found that Fe_3_O_4_ nanocrystallites transformed into *γ*-Fe_2_O_3_ nanocrystallites using Fe(NO_3_)_3_ solution treatment [30]. Therefore, it is judged that the magnetic compound for the as-prepared samples is *γ*-Fe_2_O_3_, rather than Fe_3_O_4_. Furthermore, we demonstrated the following synthesis: the precursor of FeOOH was employed as FeOOH/Mg(OH)_2_, and the resultant complex phase was transformed into *γ*-Fe_2_O_3_ crystallites by dehydration. During this process, Mg(OH)_2_ species were dissolved in the reaction medium. Such a reaction takes place below the boiling point temperature of water. When the treating solution’s temperature is lower than 70 °C, for example 40 °C, the reaction does not reach completion, leading to the formation of only a few FeOOH nanoparticles. Thus, the compositions of as-prepared samples were as follows: sample (1) contained FeOOH nanoparticles along with *γ*-Fe_2_O_3_-coated FeCl_3_·6H_2_O (*γ*-Fe_2_O_3_/FeCl_3_·6H_2_O) nanoparticles, which correspond to the smaller and larger particles in sample (1) (See Figure 2). With a steady increase in temperature, this reaction progressed towards completion. At this stage, *γ*-Fe_2_O_3_ phase increased, but FeOOH phase decreased. Consequently, magnetization enhanced from samples (1) to (3).

When the temperature reached 70 °C and increased further, the as-prepared samples were obtained in the form of pure *γ*-Fe_2_O_3_/FeCl_3_·6H_2_O nanoparticles. Since the as-prepared samples (3), and (5) contained *γ*-Fe_2_O_3_ and FeCl_3_·6H_2_O phase, we infer that the magnetization of samples may be related to the ratio between the two phases [22]. It is noticed that though EDS measurements are usually not very sensitive to oxygen content, the ratio between Fe and Cl elements is independent on the oxygen content. So using the measured atomic percentages of Fe and Cl (a_Fe_ and a_Cl_), the molar percentages of Fe_2_O_3_ (y_Fe_) and FeCl_3_·6H_2_O compounds (y_Cl_) could be estimated by the following formulae:

(1) $$\begin{array}{l} y_{\mathrm{Fe}}=\frac{\left(a_{\mathrm{Fe}}-a_{\mathrm{Cl}}/3\right)/2}{\left(a_{\mathrm{Fe}}-a_{\mathrm{Cl}}/3\right)/2+a_{\mathrm{Cl}}/3}\\ y_{\mathrm{Cl}}=\frac{a_{\mathrm{Cl}}/3}{\left(a_{\mathrm{Fe}}-a_{\mathrm{Cl}}/3\right)/2+a_{\mathrm{Cl}}/3} \end{array}.$$

Here, y*_i_* is the molar percentage of *i* compound, in samples (3) and (5), and it can be obtained from the values of a_Fe_ and a_Cl_, which were previously measured by EDS and XPS analyses (see Table 2). The results of y*_i_* are enlisted in Table 4. As a consequence, the mass fraction percentage (z*_i_*) and the volume fraction percentage (*ϕ_i_*) of each compound in respective samples can be deduced from the following formulae:

(2) $$z_{i}=\frac{y_{i}A_{i}}{\sum y_{i}A_{i}}\times 100$$

and

(3) $$\phi_{i}=\frac{z_{i}/\rho_{i}}{\sum z_{i}/\rho_{i}}\times 100.$$

**Figure 6:**
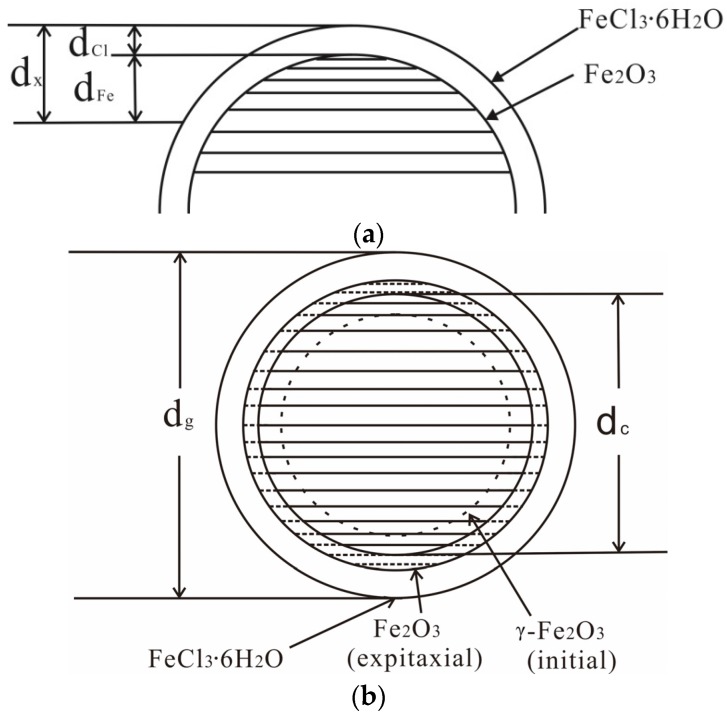
Schematic diagram of the XPS measurement’s region, d_x_, which is the depth detected by XPS (**a**). Schematic model of nanoparticle structure in samples (3) and (5) (**b**).

Here, A*_i_* and *ρ_i_* are the molar mass and the density of *i* compound, respectively. Accordingly, z*_i_* and *ϕ_i_* values of each compound in samples (3) and (5) were calculated using the values of y*_i_*, the molar mass and density of *γ*-Fe_2_O_3_ and FeCl_3_·6H_2_O (See Table 4). By referring to the data presented in Table 4, we infer that FeCl_3_·6H_2_O/Fe_2_O_3_ volume ratio (*ϕ*_Cl_/*ϕ*_Fe_) obtained for each sample by XPS was much larger than that obtained by EDS. It is well-known that EDS information is obtained from signal depths that largely exceed the dimensions of nanoparticles, whereas XPS information is obtained from the surface to a depth of approximately 3*λ* (*λ* = 1.27 nm for Fe2P electrons) [31,32]. As Figure 6a shows, EDS results depict an average *ϕ*_Cl_/*ϕ*_Fe_ across the entire particle, whereas XPS results depict the ratio of nanoparticles’ surface: d_x_ is the depth measured by XPS; d_Cl_ is the thickness of FeCl_3_·6H_2_O surface layer and d_Fe_ is Fe_2_O_3_ region probed. Thus, the difference in *ϕ*_Cl_/*ϕ*_Fe_ values computed from EDS and XPS results indicates that FeCl_3_·6H_2_O is formed outside the Fe_2_O_3_ phase [33] in samples (3) and (5).

In this experiment, the measured results of XPS indicate that *ϕ*_Cl_/*ϕ*_Fe_ value of sample (5) is greater than that of sample (3). As shown in Figure 6a, the depth probed by XPS (d_x_) can be regarded as constant, so this difference in *ϕ*_Cl_/*ϕ*_Fe_ values indicates that FeCl_3_·6H_2_O surface layer (d_Cl_) of sample (5) was thicker than that of sample (3). Therefore, FeCl_3_·6H_2_O content in as-prepared samples increased with an increase in the temperature of the treating solution. However, the measured result obtained by EDS is opposite to that obtained by XPS. Therefore, the *ϕ*_Cl_/*ϕ*_Fe_ value obtained from the measured results of EDS is smaller for sample (5) than for sample (3). Thus, we conclude that Fe_2_O_3_ content in as-prepared samples would increase steadily with an increase in temperature. Let $V_{Fe}^{\left(3\right)}$ and $V_{Fe}^{\left(5\right)}$ represent Fe_2_O_3_ volume, while $V_{Cl}^{\left(3\right)}$ and $V_{Cl}^{\left(5\right)}$ represent FeCl_3_·6H_2_O volume in samples (3) and (5), respectively. Thus, we deduce the following expressions:

(4) $$\begin{array}{l} V_{Fe}^{\left(5\right)}=V_{Fe}^{\left(3\right)}+\Delta V_{Fe}\\ V_{Cl}^{\left(5\right)}=V_{Cl}^{\left(3\right)}+\Delta V_{Cl} \end{array}$$

where ∆*V*_Fe_ and ∆*V*_Cl_ are incremental contents of Fe_2_O_3_ and FeCl_3_·6H_2_O in samples (5) and (3), respectively. The results measured by EDS help us deduce the following expression: $\phi_{Fe}^{\left(5\right)}/\phi_{Cl}^{\left(5\right)}>\phi_{Fe}^{\left(3\right)}/\phi_{Cl}^{\left(3\right)}$, where $\phi_{Fe}^{\left(5\right)}$, $\phi_{Cl}^{\left(5\right)}$, and $\phi_{Fe}^{\left(3\right)}$ $\phi_{Cl}^{\left(3\right)}$ are volume fraction percentages of Fe_2_O_3_ and FeCl_3_·6H_2_O phases in samples (5) and (3), respectively. Using the expression $\phi_{Fe}/\phi_{Cl}=V_{Fe}/V_{Cl}$, we proved that $\Delta V_{Fe}/\Delta V_{Cl}>V_{Fe}^{\left(3\right)}/V_{Cl}^{\left(3\right)}(=\phi_{Fe}^{\left(3\right)}/\phi_{Cl}^{\left(3\right)})$. Experimental results indicate that the value of $\phi_{Fe}^{\left(3\right)}/\phi_{Cl}^{\left(3\right)}$ is more than unity, so $\Delta V_{Fe}/\Delta V_{Cl}$ is more than unity. Thus, compared with sample (3), the incremental content of Fe_2_O_3_ ($\Delta V_{Fe}$) is more than the incremental content of FeCl_3_·6H_2_O ($\Delta V_{Cl}$) for sample (5). Based on these results, we proposed the following process for the formation of nanoparticles:

First, FeOOH in the precursor was subjected to dehydration, which initially led to the seeds of *γ*-Fe_2_O_3_ crystals in the solution. This reaction was accelerated and completed due to the catalytic action of FeCl_2_ treating solution; the catalytic effect of this treating solution increased as its temperature was increased by heating. Simultaneously, some Fe^2+^ in the treating solution would undergo dismutation reaction as follows: 3Fe^2+^ → 2Fe^3+^ + Fe^0^ [34,35]. Then, the resultant Fe^0^ would be oxygenated to form iron oxide in the presence of atmospheric oxygen: 4Fe^0^ + 3O_2_ → 2Fe_2_O_3_. Thus, an epitaxial Fe_2_O_3_ layer was built on initial crystallites, and FeCl_3_·6H_2_O was adsorbed onto an epitaxial layer during the precipitation process. Consequently, we synthesized *γ*-Fe_2_O_3_ based nanoparticles coated with FeCl_3_·6H_2_O. Such a chemical reaction involving the steps of dismutation and oxygenation can be written as follows:

(5) $$12{\mathrm{Fecl}}_{2}+3O_{2}\overset{\;\Delta \;}{\to }8{\mathrm{Fecl}}_{3}+2Fe_{2}O_{3}$$

A schematic model of particle structure is shown in Figure 6b. Obviously, this reaction (involving dismutation and oxygenation) would be enhanced by increasing the temperature of the treating solution. Consequently, both Fe_2_O_3_ and FeCl_3_·6H_2_O contents in as-prepared samples increased with temperature.

For the system of particles containing many phases, magnetization can be described as follows: M = *σ*<*ρ*>, where <*ρ*> is the average density of every sample, and it can be obtained as follows:

(6) $$<\rho >=\frac{\sum \phi_{i}\rho_{i}}{\sum \phi_{i}}.$$

Herein, *ϕ_i_* and *ρ_i_* are volume fraction percentage and density of *i* phase, respectively. Thus, based on *ϕ*_Fe_ and *ϕ*_Cl_ values measured by EDS and the densities of *γ*-Fe_2_O_3_ and FeCl_3_·6H_2_O, 4.90 × 10^3^ and 1.844 × 10^3^ kg/m^3^, respectively, the *ρ* value was calculated. It was found to be 4.55 × 10^3^ and 4.62 × 10^3^ kg/m^3^ for samples (3) and (5), respectively. As a consequence, the saturation magnetization (M_s_) can be obtained from *σ*_s_ and *ρ*, and it was computed to be 320.82 and 307.74 kA/m for samples (3) and (5), respectively. In addition, the magnetization can be determined as follows: M = (*ϕ*_Fe_M_Fe_ + *ϕ*_Cl_M_Cl_)/100, where *M_Fe_* and *M_Cl_* are the magnetization of *γ*-Fe_2_O_3_ and FeCl_3_·6H_2_O compounds, respectively. According to the definition of volume fraction percentage, *ϕ*_Fe_ + *ϕ*_Cl_ = 100, M can be written as follows:

(7) $$M=\frac{1}{1+\phi_{Cl}/\phi_{Fe}}(M_{Fe}-M_{Cl})+M_{Cl}.$$

*M_Fe_* and *M_Cl_* are regarded as contents. Thus, using the relations *M_Fe_* >> *M_Cl_* and *ϕ*_Fe_ >> *ϕ*_Cl_, the Formula (7) can be written simply as follows:

(8) $$M=\frac{M_{Fe}}{1+\phi_{Cl}/\phi_{Fe}}.$$

From Formula (8), we conclude that saturation magnetization (M_s_) is inversely related to *ϕ*_Cl_/*ϕ*_Fe_. Therefore, the smaller the value of *ϕ*_Cl_/*ϕ*_Fe_, the stronger would be M_s_. However, experimental results appear to be contradictory because M_s_ is lower for sample (5) than for sample (3), despite the fact that the *ϕ*_Cl_/*ϕ*_Fe_ value of the former is smaller than the latter (see Table 4). This paradox means that the apparent magnetization of as-prepared sample could be not only related to chemical compounds but also to their effective magnetic compounds. We substantiate our claim in the following paragraph. 

Surface magnetic properties become extremely important with a decrease in particle size, since a decrease in particle size leads to an increase in surface-to-volume ratio. The properties depend on the surface microstructure and the surrounding, e.g., generally because of variation in the local and exchange fields [34]. In magnetic nanoparticles, crystal symmetry breaking at the surface results in surface anisotropy. This phenomenon is more pronounced in ferrimagnets [36]. Many ramifications are associated with breaking of crystal symmetry at the surface of crystallites. One of the most important developments would be the occurrence of spin disorder in the surface layer [37,38]. With a thickness of 0.3–1.0 nm, the disordered surface layer is similar to a magnetic “dead layer” [29]. Experimental results indicate that the grain size (d_c_) of both the samples (5) and (3) were almost the same when we compared the measured results obtained by XRD; however, the physical size (d_g_) measured by TEM is greater for the former (sample 5) than for the latter (sample 3), while the Fe_2_O_3_ content (*ϕ*_Fe_) measured by EDS is greater for the former than for the latter. Based on these results, we infer that the epitaxial Fe_2_O_3_ layer, which forms on the initial seed crystallites, may have a disordered surface layer due to the breaking of crystal symmetry. This expanse of the disordered layer is similar to the amorphous component and it does not influence XRD measurement because only the crystalline phase is detected with XRD [29]. The thickness of the disordered layer increases as the temperature of the treating solution is increased. Such a disordered layer seems to be magnetically silent, and it does not stimulate the apparent magnetization in any way [38]. The contents of both FeCl_3_·6H_2_O and epitaxial Fe_2_O_3_ in sample (5) are more than those in sample (3); however, the content of *γ*-Fe_2_O_3_ crystal, that is, the effective magnetic component is almost the same in samples (5) and (3), so the magnetization of sample (5) is weaker than that of sample (3). Accordingly, it can summarized that as the treating solution’s temperature was increased from 70 to 90 °C, the content of both FeCl_3_∙6H_2_O and the disordered Fe_2_O_3_ increased so that the magnetization behavior of as-prepared samples became weak with a steady increase in temperature.

The zero-field cooled (ZFC) and field-cooled (FC) measurements for magnetic behaviors can reveal the super paramagnetic behavior of a sample. This could be interesting to clarify possible interactions between the different magnetic phases in the sample [39], and will be performed in further work. Mӧssbauer spectroscopy may be used to distinguish *γ*-Fe_2_O_3_ from Fe_3_O_4_, since *γ*-Fe_2_O_3_ and Fe_3_O_4_ give quite a different spectrum, both above and below the Verwey transition [40]. It will be considered in further works that using Mӧssbauer spectroscopy can determine the maghemite phase in the nanoparticles.

## 5. Conclusions

Using FeOOH/Mg(OH)_2_ as a precursor, we prepared *γ*-Fe_2_O_3_ based magnetic nanoparticles in FeCl_2_ solution. In this chemical reaction, we found that the magnetization of as-prepared products had a non-monotonical variation with an increase in the temperature of treated solution (FeCl_2_). Experimental results indicate that the magnetization behavior of the as-prepared samples is not only related to the chemical compounds present in the particles, but it also governs the formation of nanoparticles and their effectively magnetic compounds. When the treating solution’s temperature was below 70 °C, for example 40–60 °C, the hydration reaction involving FeOOH species from the precursor does not reach completion. However, the *γ*-Fe_2_O_3_ crystallite core is formed initially in this reaction. As a result, the as-prepared samples contained FeOOH nanoparticles along with *γ*-Fe_2_O_3_/FeCl_3_·6H_2_O nanoparticles, and their magnetization levels were weaker. When the temperature of the treated FeCl_2_ solution was increased from 70 to 90 °C, we could obtain as-prepared samples containing only *γ*-Fe_2_O_3_/FeCl_3_·6H_2_O nanoparticles. Both Fe_2_O_3_ and FeCl_3_·6H_2_O contents increased completely with an increase in temperature. Furthermore, we infer that the FeCl_2_ treating solution, which has a catalytic effect on the dehydration of FeOOH and its subsequent transformation into *γ*-Fe_2_O_3_ seed crystals, could appear as a two-step reaction involving dismutation and oxygenation; the reaction led to the formation of FeCl_3_ and Fe_2_O_3_ as products of dismutation and oxygenation, respectively. Moreover, the contents of both FeCl_3_ and Fe_2_O_3_ would increase with an increase in temperature. In this synthesis reaction, Fe_2_O_3_ grows epitaxially on the initial seed crystals of *γ*-Fe_2_O_3_, whereas FeCl_3_ is absorbed to form FeCl_3_·6H_2_O on the outermost layer of the particles. This epitaxial Fe_2_O_3_ could have a γ-Fe_2_O_3_ phase layer and disordered surface layer. The disordered surface has the breaking of crystal symmetry, so it seems to be a magnetically silent layer. As a result, it does not have any role in the apparent magnetization of nanoparticles. As the treating solution’s temperature was increased tom 70 to 90 °C, the content of both the products, namely, FeCl_3_·6H_2_O and the disordered Fe_2_O_3_ increased sharply. Consequently, the magnetization behavior of as-prepared samples became weak with a steady increase in temperature.

## Figures and Tables

**Table 1:** Median size (*d_g_*), standard deviation (ln*σ*_g_) based on the TEM results, and grain size (d_c_) based on the XRD results for samples (3) and (5).

Samples	*d_g_* (nm)	ln*σ*_g_	d_c_ (nm)
(3)	11.2	0.3	9.0
(5)	12.0	0.3	9.0

**Table 2:** Atomic percentages (a*_i_*) of O, Fe, and Cl obtained by EDS and XPS measurements in samples (1), (3), and (5).

Samples	EDS			XPS		
	O	Fe	Cl	O	Fe	Cl
(1)	40.43	51.58	1.99	64.06	31.99	3.96
(3)	45.16	52.56	2.28	64.15	35.98	3.87
(5)	45.31	52.92	1.77	58.02	32.89	5.17

**Table 3:** Binding energies data from XPS (eV) for elements present in samples (1), (3), and (5).

Samples	O ls		Fe 2p_3/2_		Cl 2p_3/2_
(1)	529.32(P1)	530.46(P2)	709.66(P1)	711.19(P2)	198.7
(3)	529.38		709.67(P1)	711.44(P2)	198.5
(5)	529.36		709.63(P1)	711.40(P2)	198.5
Fe_2_O_3_	529.5		709.9		
FeCl_3_				711.3	199.0
FeOOH		530.1		711.5	

Note: Standard data from the NIST online database for XPS at http://www.nist.gov.

**Table 4:** Molar fraction percentages (y*_i_*), mass fraction percentages (z*_i_*), and volume fraction percentages (*ϕ_i_*) determined by (a) EDS and (b) XPS.

Samples	y_Fe_	y_Cl_	z_Fe_	z_Cl_	*ϕ*_Fe_	*ϕ*_Cl_	*ϕ*_Cl_/*ϕ*_Fe_
**a**							
**(3)**	97.14	2.85	95.27	4.73	88.41	11.59	0.13
**(5)**	97.80	2.20	96.33	3.67	90.88	9.12	0.10
**b**							
**(3)**	93.08	6.92	88.82	11.78	74.29	25.91	0.35
**(5)**	90.03	9.97	84.22	15.78	66.94	33.06	0.49
